# Supplementary material for: Preclinical systematic review of CCR5 antagonists as cerebroprotective and stroke recovery enhancing agents
Source: eLife. 2025 Apr 7;14:RP103245. doi: 10.7554/eLife.103245 (PMC11975378; doi:10.7554/eLife.103245)
Supplement: Supplementary file 1. [file elife-103245-supp1.zip › 103245SupplementaryFile1.docx]

**Supplementary file 1.** Full search strategies for MEDLINE / Embase and Web of Science.

Medline: 58

Embase: 118

Web of Science: 87

Total: 230

Total after duplicates: 166

Duplicates: 64

******************************************************************************

Embase Classic+Embase <1947 to 2022 October 24>

Ovid MEDLINE(R) ALL <1946 to October 24, 2022>

1 exp animal experimentation/ or exp models, animal/ or animals/ or exp animal population groups/ or chordata/ or vertebrates/ or exp amphibians/ or exp birds/ or exp fishes/ or exp reptiles/ or mammals/ or primates/ or eutheria/ or exp artiodactyla/ or exp carnivore/ or exp cephalopoda/ or exp cetacea/ or exp chiroptera/ or exp elephants/ or exp hyraxes/ or exp insectivora/ or exp lagomorpha/ or marsupialia/ or exp monotremata/ or exp perissodactyla/ or proboscidea mammal/ or exp rodentia/ or exp scandentia/ or exp sirenia/ or exp cingulata/ or haplorhini/ or exp strepsirhini/ or exp platyrrhini/ or exp tarsii/ or catarrhini/ or exp cercopithecidae/ or exp hylobatidae/ or hominidae/ or exp gorilla gorilla/ or exp pan paniscus/ or exp pan troglodytes/ or exp pongo/ 38941083

2 (rat or rats or animal or animals or mice or in vivo or mouse or rabbit or rabbits or murine or pig or pigs or dog or dogs or bovine or fish or vertebrate or vertebrates or cat or cats or rodent or rodents or mammal or mammals or chicken or chickens or monkey or monkeys or sheep or canine or canines or porcine or cattle or bird or birds or hamster or hamsters or primate or primates or cow or cows or chick or horse or horses or avian or avians or calf or swine or swines or xenopus or turkeys or bear or bears or frog or frogs or zebrafish or goat or goats or equine or calves or poultry or macaque or macaques or mole or moles or ovine or lamb or lambs or fishes or diptera or amphibian or amphibians or snake or snakes or ruminant or ruminants or hen or hens or piglet or piglets or feline or felines or simian or simians or laevis or trout or trouts or teleost or teleosts or salmon or salmons or seal or seals or bull or bulls or ewe or ewes or hedgehog or hedgehogs or macaca or macacas or proteus or pigeon or pigeons or bat or bats or duck or ducks or chimpanzee or chimpanzees or baboon or baboons or deer or rana or ranas or carp or carps or heifer or swallow or swallows or lizard or lizards or canis or sow or sows or cynomolgus or quail or quails or reptile or reptiles or turtle or turtles or buffalo or gerbil or gerbils or boar or boars or squirrel or squirrels or oncorhynchus or mus or toad or toads or fowl or fowls or rerio or danio or ara or aras or musculus or tadpole or tadpoles or mulatta or salmo or ram or eagle or eagles or ferret or ferrets or goldfish or catfish or whale or whales or fox or foxes or ape or apes or elephant or elephants or bos or marmoset or marmosets or cod or cods or shark or sharks or wolf or eel or eels or auratus or rattus or zebra or zebras or tilapia or tilapias or gilt or camel or camels or squid or gallus or marsupial or marsupials or vole or voles or fascicularis or ovis or salmonid or salmonids or tiger or tigers or dolphin or dolphins or robin or robins or carpio or opossum or opossums or cyprinus or salamander or salamanders or felis or mink or minks or swan or swans or norvegicus or bufo or torpedo or bass or lamprey or lampreys or sus or python or pythons or tetrapod or tetrapods or shrew or shrews or lion or lions or hog or hogs or songbird or songbirds or oreochromis or starling or starlings or caprine or carassius or owl or owls or newt or newts or papio or scrofa or hare or hares or gorilla or gorillas or flounder or flounders or goose or herring or herrings or therian or buffaloes or canary or sparrow or sparrows or microtus or octopus or troglodytes or tuna or amphibia or chinchilla or chinchillas or ide or oryzias or cervus or kangaroo or kangaroos or armadillo or armadillos or callithrix or pan troglodytes or saimiri or cichlid or cichlids or donkey or donkeys or bream or char or chars or finch or raccoon or raccoons or bothrops or anguilla or perch or cricetus or seabird or seabirds or buck or bucks or naja or coturnix or salmonids or geese or minnow or minnows or raptor or raptors or merione or meriones or rodentia or elaphus or amniote or amniotes or elasmobranch or emu or emus or peromyscus or hominid or hominids or bubalus or crotalus or gull or gulls or anas or anura or lemur or lemurs or crow or crows or camelus or gibbon or gibbons or waterfowl or parrot or parrots or eels or cob or stickleback or sticklebacks or columba or mesocricetus or ambystoma or raven or ravens or gadus or penguin or penguins or orangutan or orangutans or sturgeon or sturgeons or cuniculus or aves or virginianus or cephalopod or cephalopods or cebus or sparus or tortoise or tortoises or guttata or morhua or unguiculatus or dogfish or vulpes or mallard or mallards or apodemus or alligator or alligators or oryctolagus or llama or llamas or reindeer or mustela or duckling or ducklings or wolves or sander or amazona or zebu or badger or badgers or dove or doves or ictalurus or capra or capras or equus or camelid or camelids or poecilia or mule or mules or perciformes or salvelinus or labrax or cyprinidae or ariidae or crocodile or crocodiles or fundulus or dicentrarchus or clarias or cercopithecus or chiroptera or alpaca or alpacas or pike or pikes or paralichthys or puma or pumas or didelphis or pisces or macropus or triturus or bison or bisons or epinephelus or gasterosteus or panthera or acipenser or mackerel or mackerels or tamarin or tamarins or ostrich or anolis or vervet or vervets or wallaby or glareolus or beaver or beavers or dromedary or catus or killifish or pimephales or promelas or aotus or phoca or panda or pandas or porpoise or porpoises or myotis or yak or yaks or agkistrodon or vipera or otter or otters or turbot or turbots or squamate or carnivora or mullet or mullets or hawk or hawks or taeniopygia or seahorse or seahorses or poecilia reticulata or falcon or falcons or prosimian or prosimians or parus or perca or fingerling or fingerlings or antelope or antelopes or tupaia or passeriformes or sepia or saguinus or coyote or coyotes or pongo or meleagris or reptilia or lepus or psittacine or hagfish or warbler or warblers or russell's viper or russell's vipers or smolt or smolts or budgerigar or sardine or sardines or cavia or cavias or hyla or pleurodeles or siluriformes or great tit or great tits or guppy or bonobo or bonobos or rutilus or trichosurus or muridae or phodopus or channa or squalus or lynx or sturnus or petromyzon or vitulina or monodelphis or cuttlefish or adder or adders or lepomis or canaria or gambusia or guppies or xiphophorus or flatfish or koala or koalas or labeo or stingray or stingrays or chelonia or lampetra or spermophilus or crocodilian or passer domesticus or sciurus or artiodactyla or ranidae or corvus or necturus or platypus or canaries or bovid or lagopus or trimeresurus or gariepinus or marten or martens or drosophilidae or mugil or sunfish or porcellus or cypriniformes or alouatta or scophthalmus or anser or electrophorus or putorius or iguana or iguanas or lama or lamas or takifugu or circus or eptesicus or flycatcher or galago or galagos or trachemys or lungfish or characiformes or shorebird or shorebirds or giraffe or giraffes or micropterus or scyliorhinus or cichlidae or loligo or porcupine or porcupines or chub or chubs or solea or pleuronectes or hylidae or viperidae or echis or sorex or anchovy or lagomorph or ostriches or vulture or vultures or whitefish or araneus or jird or jirds or tern or esox or drake or drakes or elapidae or gallopavo or chordata or myodes or caretta or serinus or grouse or misgurnus or meles or blackbird or blackbirds or coregonus or bobwhite or bobwhites or heteropneustes or mammoth or mammoths or turdus or rhinella or ateles or characidae or clupea or bungarus or brill or struthio camelus or sloth or sloths or pteropus or sculpin or anthropoids or pollock or pollocks or morone or pan paniscus or litoria or chipmunk or chipmunks or balaenoptera or marmota or melopsittacus or hyrax or lemming or lemmings or halibut or hylobates or lates or caiman or caimans or sigmodon or stenella or barbel or barbels or sterna or parakeet or parakeets or phocoena or leptodactylus or canidae or buteo or harengus or gopher or gophers or marmot or marmots or gosling or goslings or platichthys or gar or gars or sebastes or marsupialia or notophthalmus or gazelle or gazelles or insectivora or paridae or felidae or russula or galliformes or bombina or colobus or echidna or echidnas or seabass or syncerus or plaice or blue tit or blue tits or pagrus or catfishes or cetacea or barbus or cygnus or ficedula or chamois or colubridae or perches or coelacanth or fitch or urodela or cynops or martes or halichoerus or aix or salmonidae or leuciscus or magpie or magpies or silurus or whiting or whitings or anseriformes or colinus or rhea or chlorocebus or octodon or acinonyx or mouflon or mouflons or ibex or tetraodon or bufonidae or equidae or jackal or cephalopoda or dendroaspis or glama or muskrat or muskrats or sable or sables or wildebeest or streptopelia or albifrons or vespertilionidae or woodpecker or woodpeckers or muntjac or muntjacs or archosaur or branta or cricetulus or megalobrama or poeciliidae or desmodus or snakehead or snakeheads or tench or teal or teals or bandicoot or bandicoots or apteronotus or phyllostomidae or crocidura or buzzard or buzzards or larimichthys or cercocebus or pipistrellus or erithacus or impala or impalas or rousettus or haddock or haddocks or tinca or ratite or calidris or cynoglossus or hypophthalmichthys or bullock or bullocks or dromedaries or alectoris or filly or salamandra or cingulata or bitis or grus or ammodytes or macaw or macaws or hypoleuca or sapajus or cyprinodontiformes or hippopotamus or pelophylax or capybara or capybaras or weasel or weasels or cairina or cynomys or lutra or cockatoo or cockatoos or lachesis or lagomorpha or rupicapra or daboia or orang utan or orang utans or platyrrhini or charadriiformes or micrurus or psittaciformes or spalax or loris or mustelidae or sylvilagus or vitticeps or cockatiel or mustelus or cottus or erythrocebus or dipodomys or platessa or callicebus or loricariidae or catostomus or cuneata or cyanistes or cyprinodon or sigmodontinae or elasmobranchii or trichechus or sauropsid or xenarthra or dormouse or perissodactyla or nautilus or cirrhinus or gulo or tragelaphus or merula or numida or sciaenidae or cerastes or sciuridae or gibbosus or octopuses or eland or elands or phyllomedusa or pogona or walrus or agamidae or leptodactylidae or ridibundus or leontopithecus or anteater or anteaters or pelodiscus or cebidae or columbianus or pelteobagrus fulvidraco or hominoidea or mandrillus or zonotrichia leucophrys or agama or gobiocypris or bearded dragon or bearded dragons or sarotherodon or talpa or discoglossus or hagfishes or sphenodon or gudgeon or amphiuma or aythya or tenrec or tenrec or hominidae or risoria or salamandridae or camelidae or columbiformes or latimeria or plover or plovers or afrotheria or falco sparverius or polecat or polecats or crotalinae or salvadora or tarsier or lucioperca or anchovies or lungfishes or terrapin or dromaius novaehollandiae or lateolabrax or eigenmannia or pelamis or theropithecus or murinae or gander or gymnotus or pseudacris or gymnophiona or gymnotiformes or laticauda or falconiformes or dugong or dugongs or pintail or pintails or rook or rooks or lasiurus or catshark or catsharks or micropogonias or red junglefowl or paddlefish or ophiophagus or hollandicus or nymphicus or pimelodidae or aepyceros or cobitidae or strigiformes or cobitis or dormice or alytes or calloselasma or guanaco or phasianidae or round goby or trichogaster or catarrhini or eelpout or eelpouts or galaxias or gaur or pungitius or suslik or susliks or flatfishes or percidae or caprinae or todarodes or osmerus or ameiurus or anthropoidea or castor canadensis or pouting or poutings or tetraodontiformes or arvicolinae or siamang or siamangs or castor fiber or nomascus or red knot or red knots or syngnathidae or iguanidae or eretmochelys or ursidae or callimico or columbidae or microhylidae or anaxyrus or menidia or pipistrelle or greylag or pipidae or scandentia or bowfin or bowfins or dendrobatidae or zenaida or bushbaby or harrier or harriers or macropodidae or pygerythrus or clupeidae or odorrana or corvidae or jerboa or jerboas or canutus or hylobatidae or clupeiformes or great cormorant or great cormorants or scorpaeniformes or chondrostean or garfish or proboscidea or psetta or diapsid or serotinus or tetrao or walruses or carcharhiniformes or leucoraja or pumpkinseed or dosidicus or acipenseriformes or daubentonii or emberizidae or gadiformes or hyraxes or stizostedion or wolverine or wolverines or lissotriton or acanthurus or centrarchidae or gloydius or laurasiatheria or limosa or psittacula or leporidae or proteidae or zander or zanders or arapaima or bagridae or cyprinodontidae or mithun or pandion or jackdaw or jackdaws or procyonidae or carus or jaculus or salmoniformes or common sole or common soles or protobothrops or calamita or brachyteles or trionyx or turdidae or boidae or luscinia or pugnax or euarchontoglires or saithe or saithes or symphalangus or aardvark or aardvarks or oystercatcher or oystercatchers or arius or corydoras or poacher or poachers or aurochs or cebuella or crecca or lemuridae or sirenia or lemmus or perdix or glires or lepidosaur or muskox or deinagkistrodon or pholidota or holocephali or cercopithecinae or clariidae or agapornis or doryteuthis or tyrannidae or dicroglossidae or godwit or godwits or monedula or pongidae or atheriniformes or colobinae or lophocebus or atelidae or cottidae or leucopsis or acanthuridae or didelphimorphia or elver or elvers or lapponica or dermoptera or european hake or european hakes or gerbillinae or banteng or hartebeest or hartebeests or hogget or haematopus or anguis fragilis or grey heron or grey herons or blue whiting or blue whitings or furnariidae or macrovipera or esocidae or lapwing or lapwings or mylopharyngodon or wallabia or beloniformes or potoroo or potoroos or athene noctua or pleuronectidae or bushbabies or muscicapidae or alligatoridae or fuligula or bush baby or guineafowl or spoonbill or spoonbills or viverridae or catostomidae or zebrafishes or ibexes or vendace or estrildidae or monotremata or sepiella or ambystomatidae or shelduck or shelducks or treeshrew or treeshrews or hoplobatrachus or pochard or hoolock or hoolocks or lynxes or antilope or antilopes or blackbuck or blackbucks or cricetinae or paramisgurnus or skylark or skylarks or soleidae or allobates or northern wheatear or northern wheatears or pitheciidae or takin or theria or vanellus or galaxiidae or lorisidae or ostralegus or palaeognathae or stone loach or alauda or callitrichinae or caniformia or duttaphrynus or ictaluridae or osteoglossiformes or poultries or curema or ruddy turnstone or ruddy turnstones or sheatfish or sunfishes or centropomidae or hemachatus or platalea or thamnophilidae or song thrush or atherinopsidae or siluridae or tadorna or chroicocephalus or ermine or ermines or gavialis or ruff or tupaiidae or diprotodontia or hyaenidae or antilopinae or crocodylidae or herpestidae or hippopotamidae or northern shoveler or round gobies or cheirogaleidae or indriidae or fundulidae or pythonidae or rhynchocephalia or anodorhynchus or red-backed shrike or red-backed shrikes or triakidae or phalangeridae or aoudad or boreoeutheria or eurasianjay or eurasian jays or feliformia or haplorhini or osteoglossidae or paenungulata or struthioniformes or ferina or sanderling or sanderlings or spheniscidae or cuttlefishes or cygnet or dasycneme or gadwall or gadwalls or pelobates fuscus or wryneck or wrynecks or afrosoricida or culaea or dover sole or dover soles or paralichthyidae or passeridae or osteolaemus or song thrushes or bluethroat or bluethroats or hydrophiidae or megrim or mephitidae or strepsirhini or tomistoma or epidalea or osmeriformes or bush babies or tarsiiform or atelinae or bufotes or eurasian coot or eurasian coots or galagidae or geopelia or philomachus or tubulidentata or bombinatoridae or pelobatidae or tachysurus or ailuridae or woodlark or woodlarks or alcelaphinae or redshank or redshanks or salientia or sand smelt or sand smelts or woodmice or woodmouse or dasyproctidae or eurasian wigeon or eurasian wigeons or garganey or garganeys or lemon sole or lemon soles or common dab or common dabs or graylag or graylags or leucorodia or osphronemidae or bewickii or common moorhen or common moorhens or decapodiformes or gobbler or gobblers or odontophoridae or paddlefishes or eutheria or salmonine or esociformes or eurasian woodcock or eurasian woodcocks or european smelt or european smelts or goldfishes or tenches or tyranni or common chaffinch or common chaffinchs or common redstart or common redstarts or common roach or common roachs or great knot or great knots or potoroidae or alytidae or coregonine or dipteral or leveret or poeciliopsis gracilis or amphiumidae or batrachoidiformes or bighead goby or heteropneustidae or lullula or norway pout or norway pouts or sipunculida or dogfishes or sebastidae or tarsiidae or alethinophidia or common nase or common nases or common sandpiper or common sandpipers or eurasian blackcap or eurasian blackcaps or pterocnemia or syngnathiformes or common chaffinches or eupleridae or octopodiformes or phascolarctidae or scophthalmidae or starry smooth-hound or starry smooth-hounds or whitefishes or cuniculidae or european sprat or european sprats or rosy bitterling or rosy bitterlings or common dace or common daces or lesser weever or lesser weevers or scaldfish or water rail or water rails or alouattinae or centrarchiformes or common whitethroat or common whitethroats or gavialidae or grey gurnard or grey gurnards or lateolabracidae or rheiformes or tubgurnard or tub gurnards or common chiffchaff or common chiffchaffs or garfishes or lesser whitethroat or lesser whitethroats or myoxidae or seabasses or spariformes or umbridae or yellow boxfish or anabantiformes or aotidae or common bleak or common bleaks or common rudd or common rudds or greater pipefish or hapale or nandiniidae or stone loaches or whinchat or whinchats or acanthuriformes or brotula barbata or common ling or common lings or common roaches or cottonrat or cottonrats or douroucoulis or dromaiidae or fitches or fitchew or galaxiiformes or laprine or saimiriinae or solenette or tarsii or tompot blenny or common dragonet or common dragonets or longspined bullhead or longspined bullheads or monotremate or monotremates or pempheriformes or perdicinae or presbytini or smegmamorpha or bighead gobies or carangaria incertae sedis or coiidae or fivebeard rockling or foulmart or foumart or grasskeet or greater pipefishes or ibices or millionfish or muguliformes or norwegian topknot or peewit or red sea sailfin tang or rupicapras or sheatfishes or tompot blennies or twait shad or yellow boxfishes).tw. 14176107

3 medline.st. 29904626

4 2 not 3 8398417

5 1 or 4 40352035

6 "Receptors, CCR5"/ 18466

7 (cc chemokine receptor* 5 or cc-ckr5 or ccr5 or cd195 antigen* or ckr5 receptor*).tw,kf. 22382

8 exp CCR5 Receptor Antagonists/ 8211

9 C-C chemokine receptor type 5.tw,kf. 434

10 (md6p741w8a or maraviroc or selzentry or Celsentri or uk 427,857 or uk-427,857 or uk427,857).tw,kf. 3408

11 Vicriviroc.mp. 728

12 (Aplaviroc or ancriviroc or aplaviroc or leronlimab or mavorixafor).mp. 866

13 INCB009471.mp. 2

14 TBR 652.mp. 37

15 Pro 140.mp. 297

16 HGS004.mp. 15

17 6 or 7 or 8 or 9 or 10 or 11 or 12 or 13 or 14 or 15 or 16 33160

18 5 and 17 23661

19 exp cerebrovascular disorders/ or stroke/ 1287736

20 stroke*.tw,kf. 792358

21 transient isch?em* attack*.tw,kf. 42566

22 brain injur*.tw,kf. 199884

23 ((brain or cereb* or intracereb*) adj2 (isch?em* or infarct* or h?emorrhag*)).tw,kf. 261843

24 or/19-23 1714778

25 18 and 24 373

**26** **25 use medall** **58**

27 exp animal experiment/ or exp animal model/ or exp experimental animal/ or exp transgenic animal/ or exp male animal/ or exp female animal/ or exp juvenile animal/ or animal/ or chordata/ or vertebrate/ or tetrapod/ or exp fish/ or amniote/ or exp amphibia/ or mammal/ or exp reptile/ or exp sauropsid/ or therian/ or exp monotreme/ or placental mammal/ or exp marsupial/ or Euarchontoglires/ or exp Afrotheria/ or exp Boreoeutheria/ or exp Laurasiatheria/ or exp Xenarthra/ or primate/ or exp Dermoptera/ or exp Glires/ or exp Scandentia/ or Haplorhini/ or exp prosimian/ or simian/ or exp tarsiiform/ or Catarrhini/ or exp Platyrrhini/ or ape/ or exp Cercopithecidae/ or hominid/ or exp hylobatidae/ or exp chimpanzee/ or exp gorilla/ or exp orang utan/ or exp cephalopod/ 14616185

28 (rat or rats or animal or animals or mice or "in vivo" or mouse or rabbit or rabbits or murine or pig or pigs or dog or dogs or bovine or fish or vertebrate or vertebrates or cat or cats or rodent or rodents or mammal or mammals or chicken or chickens or monkey or monkeys or sheep or canine or canines or porcine or cattle or bird or birds or hamster or hamsters or primate or primates or cow or cows or chick or horse or horses or avian or avians or calf or swine or swines or xenopus or turkeys or bear or bears or frog or frogs or zebrafish or goat or goats or equine or calves or poultry or macaque or macaques or mole or moles or ovine or lamb or lambs or fishes or diptera or amphibian or amphibians or snake or snakes or ruminant or ruminants or hen or hens or piglet or piglets or feline or felines or simian or simians or laevis or trout or trouts or teleost or teleosts or salmon or salmons or seal or seals or bull or bulls or ewe or ewes or hedgehog or hedgehogs or macaca or macacas or proteus or pigeon or pigeons or bat or bats or duck or ducks or chimpanzee or chimpanzees or baboon or baboons or deer or deers or rana or ranas or carp or carps or heifer or swallow or swallows or lizard or lizards or canis or sow or sows or cynomolgus or quail or quails or reptile or reptiles or turtle or turtles or buffalo or gerbil or gerbils or boar or boars or squirrel or squirrels or oncorhynchus or mus or toad or toads or fowl or fowls or rerio or danio or ara or aras or musculus or tadpole or tadpoles or mulatta or salmo or ram or eagle or eagles or ferret or ferrets or goldfish or catfish or whale or whales or fox or foxes or ape or apes or elephant or elephants or bos or marmoset or marmosets or cod or cods or shark or sharks or wolf or eel or eels or auratus or rattus or zebra or zebras or tilapia or tilapias or gilt or camel or camels or squid or gallus or marsupial or marsupials or vole or voles or fascicularis or ovis or salmonid or salmonids or tiger or tigers or dolphin or dolphins or robin or robins or carpio or opossum or opossums or cyprinus or salamander or salamanders or felis or mink or minks or swan or swans or norvegicus or bufo or torpedo or bass or lamprey or lampreys or sus or python or pythons or tetrapod or tetrapods or shrew or shrews or lion or lions or hog or hogs or songbird or songbirds or oreochromis or starling or starlings or caprine or carassius or owl or owls or newt or newts or papio or scrofa or hare or hares or gorilla or gorillas or flounder or flounders or goose or herring or herrings or therian or buffaloes or canary or sparrow or sparrows or microtus or octopus or troglodytes or tuna or amphibia or chinchilla or chinchillas or ide or oryzias or cervus or kangaroo or kangaroos or armadillo or armadillos or callithrix or "pan troglodytes" or saimiri or cichlid or cichlids or donkey or donkeys or bream or char or chars or finch or raccoon or raccoons or bothrops or anguilla or perch or cricetus or seabird or seabirds or buck or bucks or naja or coturnix or salmonids or geese or minnow or minnows or raptor or raptors or merione or meriones or rodentia or elaphus or amniote or amniotes or elasmobranch or emu or emus or peromyscus or hominid or hominids or bubalus or crotalus or gull or gulls or anas or anura or lemur or lemurs or crow or crows or camelus or gibbon or gibbons or waterfowl or parrot or parrots or eels or cob or stickleback or sticklebacks or columba or mesocricetus or ambystoma or raven or ravens or gadus or penguin or penguins or orangutan or orangutans or sturgeon or sturgeons or cuniculus or aves or virginianus or cephalopod or cephalopods or cebus or sparus or tortoise or tortoises or guttata or morhua or unguiculatus or dogfish or vulpes or mallard or mallards or apodemus or alligator or alligators or oryctolagus or llama or llamas or reindeer or mustela or duckling or ducklings or wolves or sander or amazona or zebu or badger or badgers or dove or doves or ictalurus or capra or capras or equus or camelid or camelids or poecilia or mule or mules or perciformes or salvelinus or labrax or cyprinidae or ariidae or crocodile or crocodiles or fundulus or dicentrarchus or clarias or cercopithecus or chiroptera or alpaca or alpacas or pike or pikes or paralichthys or puma or pumas or didelphis or pisces or macropus or triturus or bison or bisons or epinephelus or gasterosteus or panthera or acipenser or mackerel or mackerels or tamarin or tamarins or ostrich or anolis or vervet or vervets or wallaby or glareolus or beaver or beavers or dromedary or catus or killifish or pimephales or promelas or aotus or phoca or panda or pandas or porpoise or porpoises or myotis or yak or yaks or agkistrodon or vipera or otter or otters or turbot or turbots or squamate or carnivora or mullet or mullets or hawk or hawks or taeniopygia or seahorse or seahorses or "poecilia reticulata" or falcon or falcons or prosimian or prosimians or parus or perca or fingerling or fingerlings or antelope or antelopes or tupaia or passeriformes or sepia or saguinus or coyote or coyotes or pongo or meleagris or reptilia or lepus or psittacine or hagfish or warbler or warblers or "russell s viper" or "russell s vipers" or smolt or smolts or budgerigar or sardine or sardines or cavia or cavias or hyla or pleurodeles or siluriformes or "great tit" or "great tits" or guppy or bonobo or bonobos or rutilus or trichosurus or muridae or phodopus or channa or squalus or lynx or sturnus or petromyzon or vitulina or monodelphis or cuttlefish or adder or adders or lepomis or canaria or gambusia or guppies).tw. 14111285

29 (xiphophorus or flatfish or koala or koalas or labeo or stingray or stingrays or chelonia or lampetra or spermophilus or crocodilian or "passer domesticus" or sciurus or artiodactyla or ranidae or corvus or necturus or platypus or canaries or bovid or lagopus or trimeresurus or gariepinus or marten or martens or drosophilidae or mugil or sunfish or porcellus or cypriniformes or alouatta or scophthalmus or anser or electrophorus or putorius or iguana or iguanas or lama or lamas or takifugu or circus or eptesicus or flycatcher or galago or galagos or trachemys or lungfish or characiformes or shorebird or shorebirds or giraffe or giraffes or micropterus or scyliorhinus or cichlidae or loligo or porcupine or porcupines or chub or chubs or solea or pleuronectes or hylidae or viperidae or echis or sorex or anchovy or lagomorph or ostriches or vulture or vultures or whitefish or araneus or jird or jirds or tern or esox or drake or drakes or elapidae or gallopavo or chordata or myodes or caretta or serinus or grouse or misgurnus or meles or blackbird or blackbirds or coregonus or bobwhite or bobwhites or heteropneustes or mammoth or mammoths or turdus or rhinella or ateles or characidae or clupea or bungarus or brill or "struthio camelus" or sloth or sloths or pteropus or sculpin or anthropoids or pollock or pollocks or morone or "pan paniscus" or litoria or chipmunk or chipmunks or balaenoptera or marmota or melopsittacus or hyrax or lemming or lemmings or halibut or hylobates or lates or caiman or caimans or sigmodon or stenella or barbel or barbels or sterna or parakeet or parakeets or phocoena or leptodactylus or canidae or buteo or harengus or gopher or gophers or marmot or marmots or gosling or goslings or platichthys or gar or gars or sebastes or marsupialia or notophthalmus or gazelle or gazelles or insectivora or paridae or felidae or russula or galliformes or bombina or colobus or echidna or echidnas or seabass or syncerus or plaice or "blue tit" or "blue tits" or pagrus or catfishes or cetacea or barbus or cygnus or ficedula or chamois or colubridae or perches or coelacanth or fitch or urodela or cynops or martes or halichoerus or aix or salmonidae or leuciscus or magpie or magpies or silurus or whiting or whitings or anseriformes or colinus or rhea or chlorocebus or octodon or acinonyx or mouflon or mouflons or ibex or tetraodon or bufonidae or equidae or jackal or cephalopoda or dendroaspis or glama or muskrat or muskrats or sable or sables or wildebeest or streptopelia or albifrons or vespertilionidae or woodpecker or woodpeckers or muntjac or muntjacs or archosaur or branta or cricetulus or megalobrama or poeciliidae or desmodus or snakehead or snakeheads or tench or teal or teals or bandicoot or bandicoots or apteronotus or phyllostomidae or crocidura or buzzard or buzzards or larimichthys or cercocebus or pipistrellus or erithacus or impala or impalas or rousettus or haddock or haddocks or tinca or ratite or calidris or cynoglossus or hypophthalmichthys or bullock or bullocks or dromedaries or alectoris or filly or salamandra or cingulata or bitis or grus or ammodytes or macaw or macaws or hypoleuca or sapajus or cyprinodontiformes or hippopotamus or pelophylax or capybara or capybaras or weasel or weasels or cairina or cynomys or lutra or cockatoo or cockatoos or lachesis or lagomorpha or rupicapra or daboia or "orang utan" or "orang utans" or platyrrhini or charadriiformes or micrurus or psittaciformes or spalax or loris or mustelidae or sylvilagus or vitticeps or cockatiel or mustelus or cottus or erythrocebus or dipodomys or platessa or callicebus or loricariidae or catostomus or cuneata or cyanistes or cyprinodon or sigmodontinae or elasmobranchii or trichechus or sauropsid or xenarthra or dormouse or perissodactyla or nautilus or cirrhinus or gulo or gulos or tragelaphus or merula or numida or sciaenidae or cerastes or sciuridae or gibbosus or octopuses or eland or elands or phyllomedusa or pogona or walrus or agamidae or leptodactylidae or ridibundus or leontopithecus or anteater or anteaters or pelodiscus or cebidae or columbianus or "pelteobagrus fulvidraco" or hominoidea or mandrillus or "zonotrichia leucophrys" or agama or gobiocypris or "bearded dragon" or "bearded dragons" or sarotherodon or talpa or discoglossus or hagfishes or sphenodon or gudgeon or amphiuma or aythya or tenrec or tenrec or hominidae or risoria or salamandridae or camelidae or columbiformes or latimeria or plover or plovers or afrotheria or "falco sparverius" or polecat or polecats or crotalinae or salvadora or tarsier or lucioperca or anchovies or lungfishes or terrapin or "dromaius novaehollandiae" or lateolabrax or eigenmannia or pelamis or theropithecus or murinae or gander or gymnotus or pseudacris or gymnophiona or gymnotiformes or laticauda or falconiformes or dugong or dugongs or pintail or pintails or rook or rooks or lasiurus or catshark or catsharks or micropogonias or "red junglefowl" or paddlefish or ophiophagus or hollandicus or nymphicus or pimelodidae or aepyceros or cobitidae or strigiformes or cobitis or dormice or alytes or calloselasma or guanaco or guanacos or phasianidae or "round goby" or trichogaster or catarrhini or eelpout or eelpouts or galaxias or gaur or pungitius or suslik or susliks or flatfishes or percidae or caprinae or todarodes or osmerus or ameiurus or anthropoidea or "castor canadensis" or pouting or poutings or tetraodontiformes or arvicolinae or siamang or siamangs or "castor fiber" or nomascus or "red knot" or "red knots" or syngnathidae or iguanidae or eretmochelys or ursidae or callimico or columbidae or microhylidae or anaxyrus or menidia or pipistrelle or greylag or pipidae or scandentia or bowfin or bowfins or dendrobatidae or zenaida or bushbaby or harrier or harriers or macropodidae or pygerythrus or clupeidae or odorrana or corvidae or jerboa or jerboas or canutus or hylobatidae or clupeiformes or "great cormorant" or "great cormorants" or scorpaeniformes or chondrostean or garfish or proboscidea or psetta or diapsid or serotinus or tetrao or walruses or carcharhiniformes or leucoraja or pumpkinseed or dosidicus or acipenseriformes or daubentonii or emberizidae or gadiformes or hyraxes or stizostedion or wolverine or wolverines or lissotriton or acanthurus or centrarchidae or gloydius or laurasiatheria or limosa or psittacula or leporidae or proteidae or zander or zanders or arapaima or bagridae or cyprinodontidae or mithun or pandion or jackdaw or jackdaws or procyonidae or carus or jaculus or salmoniformes or "common sole" or "common soles" or protobothrops or calamita or brachyteles or trionyx or turdidae or boidae or luscinia or pugnax or euarchontoglires or saithe or saithes or symphalangus or aardvark).tw. 272058

30 (aardvarks or oystercatcher or oystercatchers or arius or corydoras or poacher or poachers or aurochs or cebuella or crecca or lemuridae or sirenia or lemmus or perdix or glires or lepidosaur or muskox or deinagkistrodon or pholidota or holocephali or cercopithecinae or clariidae or agapornis or doryteuthis or tyrannidae or dicroglossidae or godwit or godwits or monedula or pongidae or atheriniformes or colobinae or lophocebus or atelidae or cottidae or leucopsis or acanthuridae or didelphimorphia or elver or elvers or lapponica or dermoptera or "european hake" or "european hakes" or gerbillinae or banteng or hartebeest or hartebeests or hogget or haematopus or "anguis fragilis" or "grey heron" or "grey herons" or "blue whiting" or "blue whitings" or furnariidae or macrovipera or esocidae or lapwing or lapwings or mylopharyngodon or wallabia or beloniformes or potoroo or potoroos or "athene noctua" or pleuronectidae or bushbabies or muscicapidae or alligatoridae or fuligula or "bush baby" or guineafowl or spoonbill or spoonbills or viverridae or catostomidae or zebrafishes or ibexes or vendace or estrildidae or monotremata or sepiella or ambystomatidae or shelduck or shelducks or treeshrew or treeshrews or hoplobatrachus or pochard or hoolock or hoolocks or lynxes or antilope or antilopes or blackbuck or blackbucks or cricetinae or paramisgurnus or skylark or skylarks or soleidae or allobates or "northern wheatear" or "northern wheatears" or pitheciidae or takin or theria or vanellus or galaxiidae or lorisidae or ostralegus or palaeognathae or "stone loach" or alauda or callitrichinae or caniformia or duttaphrynus or ictaluridae or osteoglossiformes or poultries or curema or "ruddy turnstone" or "ruddy turnstones" or sheatfish or sunfishes or centropomidae or hemachatus or platalea or thamnophilidae or "song thrush" or atherinopsidae or siluridae or tadorna or chroicocephalus or ermine or ermines or gavialis or ruff or tupaiidae or diprotodontia or hyaenidae or antilopinae or crocodylidae or herpestidae or hippopotamidae or "northern shoveler" or "round gobies" or cheirogaleidae or indriidae or fundulidae or pythonidae or rhynchocephalia or anodorhynchus or "red-backed shrike" or "red-backed shrikes" or triakidae or phalangeridae or aoudad or boreoeutheria or "eurasian jay" or "eurasian jays" or feliformia or haplorhini or osteoglossidae or paenungulata or struthioniformes or ferina or sanderling or sanderlings or spheniscidae or cuttlefishes or cygnet or dasycneme or gadwall or gadwalls or "pelobates fuscus" or wryneck or wrynecks or afrosoricida or culaea or "dover sole" or "dover soles" or paralichthyidae or passeridae or osteolaemus or "song thrushes" or bluethroat or bluethroats or hydrophiidae or megrim or mephitidae or strepsirhini or tomistoma or epidalea or osmeriformes or "bush babies" or tarsiiform or atelinae or bufotes or "eurasian coot" or "eurasian coots" or galagidae or geopelia or philomachus or tubulidentata or bombinatoridae or pelobatidae or tachysurus or ailuridae or woodlark or woodlarks or alcelaphinae or redshank or redshanks or salientia or "sand smelt" or "sand smelts" or woodmice or woodmouse or dasyproctidae or "eurasian wigeon" or "eurasian wigeons" or garganey or garganeys or "lemon sole" or "lemon soles" or "common dab" or "common dabs" or graylag or graylags or leucorodia or osphronemidae or bewickii or "common moorhen" or "common moorhens" or decapodiformes or gobbler or gobblers or odontophoridae or paddlefishes or eutheria or salmonine or esociformes or "eurasian woodcock" or "eurasian woodcocks" or "european smelt" or "european smelts" or goldfishes or tenches or tyranni or "common chaffinch" or "common chaffinchs" or "common redstart" or "common redstarts" or "common roach" or "common roachs" or "great knot" or "great knots" or potoroidae or alytidae or coregonine or dipteral or leveret or "poeciliopsis gracilis" or amphiumidae or batrachoidiformes or "bighead goby" or heteropneustidae or lullula or "norway pout" or "norway pouts" or sipunculida or dogfishes or sebastidae or tarsiidae or alethinophidia or "common nase" or "common nases" or "common sandpiper" or "common sandpipers" or "eurasian blackcap" or "eurasian blackcaps" or pterocnemia or syngnathiformes or "common chaffinches" or eupleridae or octopodiformes or phascolarctidae or scophthalmidae or "starry smooth-hound" or "starry smooth-hounds" or whitefishes or cuniculidae or "european sprat" or "european sprats" or "rosy bitterling" or "rosy bitterlings" or "common dace" or "common daces" or "lesser weever" or "lesser weevers" or scaldfish or "water rail" or "water rails" or alouattinae or centrarchiformes or "common whitethroat" or "common whitethroats" or gavialidae or "grey gurnard" or "grey gurnards" or lateolabracidae or rheiformes or "tub gurnard" or "tub gurnards" or "common chiffchaff" or "common chiffchaffs" or garfishes or "lesser whitethroat" or "lesser whitethroats" or myoxidae or seabasses or spariformes or umbridae or "yellow boxfish" or anabantiformes or aotidae or "common bleak" or "common bleaks" or "common rudd" or "common rudds" or "greater pipefish" or hapale or nandiniidae or "stone loaches" or whinchat or whinchats or acanthuriformes or "brotula barbata" or "common ling" or "common lings" or "common roaches" or cottonrat or cottonrats or douroucoulis or dromaiidae or fitches or fitchew or galaxiiformes or laprine or saimiriinae or solenette or tarsii or "tompot blenny" or "common dragonet" or "common dragonets" or "longspined bullhead" or "longspined bullheads" or monotremate or monotremates or pempheriformes or perdicinae or presbytini or smegmamorpha or "bighead gobies" or "carangaria incertae sedis" or coiidae or "fivebeard rockling" or foulmart or foumart or grasskeet or "greater pipefishes" or ibices or millionfish or muguliformes or "norwegian topknot" or peewit or "red sea sailfin tang" or rupicapras or sheatfishes or "tompot blennies" or "twait shad" or "yellow boxfishes").tw. 21723

31 or/27-30 18002118

32 exp chemokine receptor CCR5 antagonist/ 6682

33 (cc chemokine receptor* 5 or cc-ckr5 or ccr5 or cd195 antigen* or ckr5 receptor* or C-C chemokine receptor type 5).tw. 22155

34 (md6p741w8a or maraviroc or selzentry or Celsentri or uk 427,857 or uk-427,857 or uk427,857).tw. 3329

35 Vicriviroc.mp. 728

36 (Aplaviroc or ancriviroc or aplaviroc or leronlimab or mavorixafor).mp. 866

37 INCB009471.mp. 2

38 TBR 652.mp. 37

39 Pro 140.mp. 297

40 HGS004.mp. 15

41 chemokine receptor CCR5/ 12698

42 or/32-41 31956

43 31 and 42 10275

44 exp cerebrovascular disease/ or exp cerebrovascular accident/ 1207681

45 stroke*.tw. 771477

46 ((brain or cereb* or intracereb*) adj2 (isch?em* or infarct* or h?emorrhag*)).tw. 247502

47 (transient isch?em* attack* or brain injur*).tw. 231020

48 44 or 45 or 46 or 47 1675247

49 43 and 48 172

**50** **49 use emczd** **118**

51 26 or 50 176

******************************************************************************

**Web of Science – October 25, 2022**

# Web of Science Search Strategy (v0.1)

# Entitlements:

- WOS.SSCI: 1900 to 2022

- WOS.AHCI: 1975 to 2022

- WOS.ISTP: 1990 to 2022

- WOS.ESCI: 2005 to 2022

- WOS.SCI: 1900 to 2022

- WOS.ISSHP: 1990 to 2022

# Searches:

1: CCR5 (Topic) Results: 11678

2: TS=(cc chemokine receptor* 5) OR TS=(C-C chemokine receptor type 5) Results: 1796

3: TS=(cc-ckr5) Results: 9

4: TS=(Vicriviroc) Results: 158

5: TS=(((md6p741w8a OR maraviroc OR selzentry OR Celsentri ))) Results: 1542

6: ALL=( (Aplaviroc or ancriviroc or aplaviroc or leronlimab or mavorixafor)) Results: 97

7: (((TS=(INCB009471)) OR TS=(TBR 652)) OR TS=( Pro 140)) OR TS=(HGS004) Results: 972

8: #7 OR #6 OR #5 OR #4 OR #3 OR #2 OR #1 Results: 14241

9: (TS=(stroke*)) OR TS=("transient isch?em* attack*") Results: 430668

10: TS=((brain or cereb* or intracereb*) NEAR/2 ischem*) Results: 77919

11: TS=(((brain or cereb* or intracereb*) NEAR/2 ischaem*)) Results: 5389

12: TS=(((brain or cereb* or intracereb*) NEAR/2 hemorrhag*)) Results: 33959

13: TS=(((brain or cereb* or intracereb*) NEAR/2 haemorrhag*)) Results: 4867

14: TS="cerebrovascular disorder*" Results: 3993

15: TS="brain injur*" Results: 121601

16: #15 OR #14 OR #13 OR #12 OR #11 OR #10 OR #9 Results: 591495

17: #8 AND #16 Results: 136

18: TS=(rat OR rats OR animal OR animals OR mice OR "in vivo" OR mouse OR rabbit OR rabbits OR murine OR pig OR pigs OR dog OR dogs OR bovine OR fish OR vertebrate OR vertebrates OR cat OR cats OR rodent OR rodents OR mammal OR mammals OR chicken OR chickens OR monkey OR monkeys OR sheep OR canine OR canines OR porcine OR cattle OR bird OR birds OR hamster OR hamsters OR primate OR primates OR cow OR cows OR chick OR horse OR horses OR avian OR avians OR calf OR swine OR swines OR xenopus OR turkeys OR bear OR bears OR frog OR frogs OR zebrafish OR goat OR goats OR equine OR calves OR poultry OR macaque OR macaques OR mole OR moles OR ovine OR lamb OR lambs OR fishes OR diptera OR amphibian OR amphibians OR snake OR snakes OR ruminant OR ruminants OR hen OR hens OR piglet OR piglets OR feline OR felines OR simian OR simians OR laevis OR trout OR trouts OR teleost OR teleosts OR salmon OR salmons OR seal OR seals OR bull OR bulls OR ewe OR ewes OR hedgehog OR hedgehogs OR macaca OR macacas OR proteus OR pigeon ORpigeons OR bat OR bats OR duck OR ducks OR chimpanzee OR chimpanzees OR baboon OR baboons OR deer OR rana OR ranas OR carp OR carps OR heifer OR swallow OR swallows OR lizard OR lizards OR canis OR sow OR sows OR cynomolgus OR quail OR quails OR reptile OR reptiles OR turtle OR turtles OR buffalo OR gerbil OR gerbils OR boar OR boars OR squirrel OR squirrels OR oncorhynchus OR mus OR toad OR toads OR fowl OR fowls OR rerio OR danio OR ara OR aras OR musculus OR tadpole OR tadpoles OR mulatta OR salmo OR ram OR eagle OR eagles OR ferret OR ferrets OR goldfish OR catfish OR whale OR whales OR fox OR foxes OR ape OR apes OR elephant OR elephants OR bos OR marmoset OR marmosets OR cod OR cods OR shark OR sharks OR wolf OR eel OR eels OR auratus OR rattus OR zebra OR zebras OR tilapia OR tilapias OR gilt OR camel OR camels OR squid OR gallus OR marsupial OR marsupials OR vole OR voles OR fascicularis OR ovis OR salmonid OR salmonids OR tiger OR tigers OR dolphin OR dolphins OR robin OR robins OR carpio OR opossumOR opossums OR cyprinus OR salamander OR salamanders OR felis OR mink OR minks OR swan OR swans OR norvegicus OR bufo OR torpedo OR bass OR lamprey OR lampreys OR sus OR python OR pythons OR tetrapod OR tetrapods OR shrew OR shrews OR lion OR lions OR hogOR hogs OR songbird OR songbirds OR oreochromis OR starling OR starlings OR caprine OR carassius OR owl OR owls OR newt OR newts OR papio OR scrofa OR hare OR hares OR gorilla OR gorillas OR flounder OR flounders OR goose OR herring OR herrings OR therianOR buffaloes OR canary OR sparrow OR sparrows OR microtus OR octopus OR troglodytes OR tuna OR amphibia OR chinchilla OR chinchillas OR ide OR oryzias OR cervus OR kangaroo OR kangaroos OR armadillo OR armadillos OR callithrix OR "pan troglodytes" OR saimiri OR cichlid OR cichlids OR donkey OR donkeys OR bream OR char OR chars OR finch OR raccoon OR raccoons OR bothrops OR anguilla OR perch OR cricetus OR seabird OR seabirds OR buck OR bucks OR naja OR coturnix OR salmonids OR geese OR minnow OR minnows ORraptor OR raptors OR merione OR meriones OR rodentia OR elaphus OR amniote OR amniotes OR elasmobranch OR emu OR emus OR peromyscus OR hominid OR hominids OR bubalus OR crotalus OR gull OR gulls OR anas OR anura OR lemur OR lemurs OR crow OR crows OR camelus OR gibbon OR gibbons OR waterfowl OR parrot OR parrots OR eels OR cob OR stickleback OR sticklebacks OR columba OR mesocricetus OR ambystoma OR raven OR ravens OR gadus OR penguin OR penguins OR orangutan OR orangutans OR sturgeon OR sturgeons OR cuniculus OR aves OR virginianus OR cephalopod OR cephalopods OR cebus OR sparus OR tortoise OR tortoises OR guttata OR morhua OR unguiculatus OR dogfish OR vulpes OR mallard OR mallards OR apodemus OR alligator OR alligators OR oryctolagus OR llama OR llamas OR reindeer OR mustela OR duckling OR ducklings OR wolves OR sander OR amazona OR zebu OR badger OR badgers OR dove OR doves OR ictalurus OR capra OR capras OR equus OR camelid OR camelids OR poecilia OR mule OR mules OR perciformes OR salvelinus OR labrax OR cyprinidae OR ariidae OR crocodile OR crocodiles OR fundulus OR dicentrarchus OR clarias OR cercopithecus OR chiroptera OR alpaca OR alpacas OR pike OR pikes OR paralichthys OR puma OR pumas OR didelphis OR pisces OR macropus OR triturus OR bison OR bisons OR epinephelus OR gasterosteus OR panthera OR acipenser OR mackerel OR mackerels OR tamarin OR tamarins OR ostrich OR anolis OR vervet OR vervets OR wallaby OR glareolus OR beaver OR beavers OR dromedary OR catus OR killifish OR pimephales OR promelas OR aotus OR phoca OR panda OR pandas OR porpoise OR porpoises OR myotis OR yak OR yaks OR agkistrodon OR vipera OR otter OR otters OR turbot OR turbots OR squamate OR carnivora OR mullet OR mullets OR hawk OR hawks OR taeniopygia OR seahorse OR seahorses OR "poecilia reticulata" OR falcon OR falcons OR prosimian OR prosimians OR parus OR perca OR fingerling OR fingerlings OR antelope OR antelopes OR tupaia OR passeriformes OR sepia OR saguinus OR coyote OR coyotes OR pongo OR meleagris OR reptilia OR lepus OR psittacine OR hagfish OR warbler OR warblers OR "russell s viper" OR "russell s vipers" OR smolt OR smolts OR budgerigar OR sardine OR sardines OR cavia OR cavias OR hyla OR pleurodeles OR siluriformes OR "great tit" OR "great tits" OR guppy OR bonobo OR bonobos OR rutilus OR trichosurus OR muridae OR phodopus OR channa OR squalus OR lynx OR sturnus OR petromyzon OR vitulina OR monodelphis OR cuttlefish OR adder OR adders OR lepomis OR canaria OR gambusia OR guppies OR xiphophorus OR flatfish OR koala ORkoalas OR labeo OR stingray OR stingrays OR chelonia OR lampetra OR spermophilus OR crocodilian OR "passer domesticus" OR sciurus OR artiodactyla OR ranidae OR corvus OR necturus OR platypus OR canaries OR bovid OR lagopus OR trimeresurus OR gariepinus ORmarten OR martens OR drosophilidae OR mugil OR sunfish OR porcellus OR cypriniformes OR alouatta OR scophthalmus OR anser OR electrophorus OR putorius OR iguana OR iguanas OR lama OR lamas OR takifugu OR circus OR eptesicus OR flycatcher OR galago OR galagos OR trachemys OR lungfish OR characiformes OR shorebird OR shorebirds OR giraffe OR giraffes OR micropterus OR scyliorhinus OR cichlidae OR loligo OR porcupine OR porcupines OR chub OR chubs OR solea OR pleuronectes OR hylidae OR viperidae OR echis OR sorex OR anchovy OR lagomorph OR ostriches OR vulture OR vultures OR whitefish OR araneus OR jird OR jirds OR tern OR esox OR drake OR drakes OR elapidae OR gallopavo OR chordata OR myodes OR caretta OR serinus OR grouse OR misgurnus OR meles OR blackbird OR blackbirds OR coregonus OR bobwhite OR bobwhites OR heteropneustes OR

mammoth OR mammoths OR turdus OR rhinella OR ateles OR characidae OR clupea OR bungarus OR brill OR "struthio camelus" OR sloth OR sloths OR pteropus OR sculpin OR anthropoids OR pollock OR pollocks OR morone OR "pan paniscus" OR litoria OR chipmunk OR chipmunks OR balaenoptera OR marmota OR melopsittacus OR hyrax OR lemming OR lemmings OR halibut OR hylobates OR lates OR caiman OR caimans OR sigmodon OR stenella OR barbel OR barbels ORsterna OR parakeet OR parakeets OR phocoena OR leptodactylus OR canidae OR buteo OR harengus OR gopher OR gophers OR marmot OR marmots OR gosling OR goslings OR platichthys OR gar OR gars OR sebastes OR marsupialia OR notophthalmus OR gazelle OR gazelles OR insectivora OR paridae OR felidae OR russula OR galliformes OR bombina OR colobus OR echidna OR echidnas OR seabass OR syncerus OR plaice OR "blue tit" OR "blue tits" OR pagrus OR catfishes OR cetacea OR barbus OR cygnus OR ficedula OR chamois OR colubridae OR perches OR coelacanth OR fitch OR urodela OR cynops OR martes OR halichoerus OR aix OR salmonidae OR leuciscus OR magpie OR magpies OR silurus OR whiting OR whitings OR anseriformes OR colinus OR rhea OR chlorocebus OR octodon OR acinonyx OR mouflon OR mouflons OR ibex OR tetraodon OR bufonidae OR equidae OR jackal OR cephalopoda OR dendroaspis OR glama OR muskrat OR muskrats OR sable OR sables OR wildebeest OR streptopelia OR albifrons OR vespertilionidae OR woodpecker OR woodpeckers OR muntjac OR muntjacs OR archosaur OR branta OR cricetulus OR megalobrama OR poeciliidae OR desmodus OR snakehead OR snakeheads OR tench OR teal OR teals OR bandicoot OR bandicoots OR apteronotus OR phyllostomidae OR crocidura OR buzzard OR buzzards OR larimichthys OR cercocebus OR pipistrellus OR erithacus OR impala OR impalas OR rousettus OR haddock OR haddocks OR tinca OR ratite OR calidris OR cynoglossus OR hypophthalmichthys OR bullock OR bullocks OR dromedaries OR alectoris OR filly OR salamandra OR cingulata OR bitis OR grus OR ammodytes OR macaw OR macaws OR hypoleuca OR sapajus OR cyprinodontiformes OR hippopotamus OR pelophylax OR capybara OR capybaras OR weasel OR weasels OR cairina OR cynomys OR lutra OR cockatoo OR cockatoos OR lachesis OR lagomorpha OR rupicapra OR daboia OR "orang utan" OR "orang utans" OR platyrrhini OR charadriiformes OR micrurus OR psittaciformes OR spalax OR loris OR mustelidae OR sylvilagus OR vitticeps OR cockatiel OR mustelus OR cottus OR erythrocebus OR dipodomys OR platessa OR callicebus OR loricariidae OR catostomus OR cuneata OR cyanistes OR cyprinodon OR sigmodontinae OR elasmobranchii OR trichechus OR sauropsid OR xenarthra OR dormouse OR perissodactyla OR nautilus OR cirrhinus OR gulo OR gulos OR tragelaphus OR merula OR numidaOR sciaenidae OR cerastes OR sciuridae OR gibbosus OR octopuses OR eland OR elands OR phyllomedusa OR pogona OR walrus OR agamidae OR leptodactylidae OR ridibundus OR leontopithecus OR anteater OR anteaters OR pelodiscus OR cebidae OR columbianus OR "pelteobagrus fulvidraco" OR hominoidea OR mandrillus OR "zonotrichia leucophrys" OR agama OR gobiocypris OR "bearded dragon" OR "bearded dragons" OR sarotherodon OR talpa OR discoglossus OR hagfishes OR sphenodon OR gudgeon OR amphiuma OR aythya OR tenrec OR tenrec OR hominidae OR risoria OR salamandridae OR camelidae OR columbiformes OR latimeria OR plover OR plovers OR afrotheria OR "falco sparverius" OR polecat OR polecats OR crotalinae OR salvadora OR tarsier OR lucioperca OR anchovies OR lungfishes OR terrapin OR "dromaius novaehollandiae" OR lateolabrax OR eigenmannia OR pelamis OR theropithecus OR murinae OR gander OR gymnotus OR pseudacris OR gymnophiona OR gymnotiformes OR laticauda OR falconiformes OR dugong OR dugongs OR pintail OR pintails OR rook ORrooks OR lasiurus OR catshark OR catsharks OR micropogonias OR "red junglefowl" OR paddlefish OR eutheria OR ophiophagus OR hollandicus OR nymphicus OR pimelodidae OR aepyceros OR cobitidae OR strigiformes OR cobitis OR dormice OR alytes OR calloselasma OR guanaco OR guanacos OR phasianidae OR "round goby" OR trichogaster OR catarrhini OR eelpout OR eelpouts OR galaxias OR gaur OR pungitius OR suslik OR susliks OR flatfishes OR percidae OR caprinae OR todarodes OR osmerus OR ameiurus OR anthropoidea OR "castor canadensis" OR pouting OR poutings OR tetraodontiformes OR arvicolinae OR siamang OR siamangs OR "castor fiber" OR nomascus OR "red knot" OR "red knots" OR syngnathidae OR iguanidae OR eretmochelys OR ursidae OR callimico OR columbidae OR microhylidaeOR anaxyrus OR menidia OR pipistrelle OR greylag OR pipidae OR scandentia OR bowfin OR bowfins OR dendrobatidae OR zenaida OR bushbaby OR harrier OR harriers OR macropodidae OR pygerythrus OR clupeidae OR odorrana OR corvidae OR jerboa OR jerboas OR canutus OR hylobatidae OR clupeiformes OR "great cormorant" OR "great cormorants" OR scorpaeniformes OR chondrostean OR garfish OR proboscidea OR psetta OR diapsid OR serotinus OR tetrao OR walruses OR carcharhiniformes OR leucoraja OR pumpkinseed OR dosidicus OR acipenseriformes OR daubentonii OR emberizidae OR gadiformes OR hyraxes OR stizostedion OR wolverine OR wolverines OR lissotriton OR acanthurus OR centrarchidae OR gloydius OR laurasiatheria OR limosa OR psittacula OR leporidae OR proteidae OR zander ORzanders OR arapaima OR bagridae OR cyprinodontidae OR mithun OR pandion OR jackdaw OR jackdaws OR procyonidae OR carus OR jaculus OR salmoniformes OR "common sole" OR "common soles" OR protobothrops OR calamita OR brachyteles OR trionyx OR turdidae OR boidae OR luscinia OR pugnax OR euarchontoglires OR saithe OR saithes OR symphalangus OR aardvark OR aardvarks OR oystercatcher OR oystercatchers OR arius OR corydoras OR poacher OR poachers OR aurochs OR cebuella OR crecca OR lemuridae OR sirenia OR lemmus OR perdix OR glires OR lepidosaur OR muskox OR deinagkistrodon OR pholidota OR holocephali OR cercopithecinae OR clariidae OR agapornis OR doryteuthis OR tyrannidae OR dicroglossidae OR godwit OR godwits OR monedula OR pongidae OR atheriniformes OR colobinae OR lophocebus OR atelidae OR cottidae OR leucopsis OR acanthuridae OR didelphimorphia OR elver OR elvers OR lapponica OR dermoptera OR "european hake" OR "european hakes" OR gerbillinae OR banteng OR hartebeest OR hartebeests OR hogget OR haematopus OR "anguis fragilis" OR "grey heron" OR "grey herons" OR "blue whiting" OR "blue whitings" OR furnariidae OR macrovipera OR esocidae OR lapwing OR lapwings OR mylopharyngodon OR wallabia OR beloniformes OR potoroo OR potoroos OR "athene noctua" OR pleuronectidae OR bushbabies OR muscicapidae OR alligatoridae OR fuligula OR "bush baby" OR guineafowl OR spoonbill OR spoonbills OR viverridae OR catostomidae OR zebrafishes OR ibexes OR vendace OR estrildidae OR monotremata OR sepiella OR ambystomatidae OR shelduck OR shelducks OR treeshrew OR treeshrews OR hoplobatrachus OR pochard OR hoolock OR hoolocks OR lynxes OR antilope OR antilopes OR blackbuck OR blackbucks OR cricetinae OR paramisgurnus OR skylark OR skylarks OR soleidae OR allobates OR "northern wheatear" OR "northern wheatears" OR pitheciidae OR takin OR theria OR vanellus OR galaxiidae OR lorisidae OR ostralegus OR palaeognathae OR "stone loach" OR alauda OR callitrichinae OR caniformia OR duttaphrynus OR ictaluridae OR osteoglossiformes OR poultries OR curema OR "ruddy turnstone" OR

"ruddy turnstones" OR sheatfish OR sunfishes OR centropomidae OR hemachatus OR platalea OR thamnophilidae OR "song thrush" OR atherinopsidae OR siluridae OR tadorna OR chroicocephalus OR ermine OR ermines OR gavialis OR ruffe OR tupaiidae OR diprotodontia OR hyaenidae OR antilopinae OR crocodylidae OR herpestidae OR hippopotamidae OR "northern shoveler" OR "round gobies" OR cheirogaleidae OR indriidae OR fundulidae OR pythonidae OR rhynchocephalia OR anodorhynchus OR "red-backed shrike" OR "red-backed shrikes" OR triakidae OR phalangeridae OR aoudad OR boreoeutheria OR "eurasian jay" OR "eurasian jays" OR feliformia OR haplorhini OR osteoglossidae OR paenungulata OR struthioniformes OR ferina OR sanderling OR sanderlings OR spheniscidae OR cuttlefishes OR cygnet OR dasycneme OR gadwall OR gadwalls OR "pelobates fuscus" OR wryneck OR wrynecks OR afrosoricida OR culaea OR "dover sole" OR "dover soles" OR paralichthyidae OR passeridae OR osteolaemus OR "song thrushes" OR bluethroat OR bluethroats OR hydrophiidae OR megrim OR mephitidae OR strepsirhini OR tomistoma OR epidalea OR osmeriformes OR "bush babies" OR tarsiiform OR atelinae OR bufotes OR "eurasian coot" OR "eurasian coots" OR galagidae OR geopelia OR philomachus OR tubulidentata OR bombinatoridae OR pelobatidae OR tachysurus OR ailuridae OR woodlark OR woodlarks OR alcelaphinae OR redshank OR redshanks OR salientia OR "sand smelt" OR "sand smelts" OR woodmice OR woodmouse OR dasyproctidae OR "eurasian wigeon" OR "eurasian wigeons" OR garganey OR garganeys OR "lemon sole" OR "lemon soles" OR "common dab" OR "common dabs" OR graylag OR graylags OR leucorodia OR osphronemidae OR bewickii OR "common moorhen" OR "common moorhens" OR decapodiformes OR gobbler OR gobblers OR odontophoridae OR paddlefishes OR salmonine OR esociformes OR "eurasian woodcock" OR "eurasian woodcocks" OR "european smelt" OR "european smelts" OR goldfishes OR tenches OR tyranni OR "common chaffinch" OR "common chaffinchs" OR "common redstart" OR "common redstarts" OR "common roach" OR "common roachs" OR "great knot" OR "great knots" OR potoroidae OR alytidae OR coregonine OR dipteral OR leveret OR "poeciliopsis gracilis" OR amphiumidae OR batrachoidiformes OR "bighead goby" OR heteropneustidae OR lullula OR "norway pout" OR "norway pouts" OR sipunculida OR dogfishes OR sebastidae OR tarsiidae OR alethinophidia OR "common nase" OR "common nases" OR "common sandpiper" OR "common sandpipers" OR "eurasian blackcap" OR "eurasian blackcaps" OR pterocnemia OR syngnathiformes OR "common chaffinches" OR eupleridae OR octopodiformes OR phascolarctidae OR scophthalmidae OR "starry smooth-hound" OR "starry smooth-hounds" OR whitefishes OR cuniculidae OR "european sprat" OR "european sprats" OR "rosy bitterling" OR "rosy bitterlings" OR "common dace" OR "common daces" OR "lesser weever" OR "lesser weevers" OR scaldfish OR "water rail" OR "water rails" OR alouattinae OR centrarchiformes OR "common whitethroat" OR "common whitethroats" OR gavialidae OR "grey gurnard" OR "greygurnards" OR lateolabracidae OR rheiformes OR "tub gurnard" OR "tub gurnards" OR "common chiffchaff" OR "common chiffchaffs" OR garfishes OR "lesser whitethroat" OR "lesser whitethroats" OR myoxidae OR seabasses OR spariformes OR umbridae OR "yellow boxfish" OR anabantiformes OR aotidae OR "common bleak" OR "common bleaks" OR "common rudd" OR "common rudds" OR "greater pipefish" OR hapale OR nandiniidae OR "stone loaches" OR whinchat OR whinchats OR acanthuriformes OR "brotula barbata" OR "common ling" OR "common lings" OR "common roaches" OR cottonrat OR cottonrats OR douroucoulis OR dromaiidae OR fitches OR fitchew OR galaxiiformes OR laprine OR saimiriinae OR solenette OR tarsii OR "tompot blenny" OR "common dragonet" OR "common dragonets" OR "longspinedbullhead" OR "longspined bullheads" OR monotremate OR monotremates OR pempheriformes OR perdicinae OR presbytini OR smegmamorpha OR "bighead gobies" OR "carangaria incertae sedis" OR coiidae OR "fivebeard rockling" OR foulmart OR foumart OR grasskeet OR "greater pipefishes" OR ibices OR millionfish OR muguliformes OR "norwegian topknot" OR peewit OR "red sea sailfin tang" OR rupicapras OR sheatfishes OR "tompot blennies" OR "twait shad" OR "yellow boxfishes") Results: 10177702

19: #17 AND #18 Results: 87

**********************************************************************************
