# Supplementary material for: Preclinical systematic review of CCR5 antagonists as cerebroprotective and stroke recovery enhancing agents
Source: eLife. 2025 Apr 7;14:RP103245. doi: 10.7554/eLife.103245 (PMC11975378; doi:10.7554/eLife.103245)
Supplement: Supplementary file 2. [file elife-103245-supp2.zip › 103245SupplementaryFile2.docx]

**Supplementary file 2.** Data Extraction Forms

Study Characteristics

1. What is the year of publication?
2. What journal is the article published in?
3. Name of the first author? (Full last name, initial of first name - e.g. Smith, K)
4. Name and email of the corresponding author? (e.g. Smith K, smithk89@uottawa.ca)
5. What is the source of funding?
   1. Not Reported
   2. Government
   3. Industry
   4. Academic Institution
   5. Charity/Foundation
   6. Other
   7. Unclear
6. What country is the corresponding author from (look at corresponding address)?

Animal Model

1. What is the species?
   1. Mouse
   2. Rat
   3. Monkey
   4. Rabbit
   5. Other
2. What type of stroke is being studied?
   1. Ischemic
   2. Hemorrhagic
   3. Other
3. What type of stroke model is being used?
   1. Intraluminal suture (e.g. Filament, distal, and transient middle cerebral artery occlusion)
      1. What is the number of minutes of occlusion time (relevant to lesion severity)?
   2. Permanent middle cerebral artery occlusion (i.e. cauterization, permanent clip, permanent distal middle cerebral artery ligation)
   3. Photothrombotic
   4. Embolism
   5. Modified 3 vessel occlusion
   6. Endothelin-1
   7. Microvascular embolic
   8. L-NIO
   9. Intracerebral hemorrhagic
   10. Other (please specify)
4. What are the sexes of the animals?
   1. Male
   2. Female
   3. Male and female
   4. Not reported
5. What is the weight in grams and, if applicable, specify when the weight(s) was measured?
6. What is the age in weeks of the species?
7. Are there comorbidities in the animals have (e.g. aged, obesity, diabetes/hyperglycemia, hypertension)?
   1. Yes (please specify)
   2. No
8. What specific area of the brain was the stroke induced in?
   1. Frontal lobe
   2. Parietal lobe
   3. Temporal lobe
   4. Occipital lobe
   5. Cerebellum
   6. Thalamus
   7. Basal ganglia
   8. Other (please describe)
   9. Not reported
9. Were there any animals excluded?
   1. Yes (please explain why i.e. death, no impairment from stroke, etc.)
   2. No
10. What was the total number of animals excluded from the study?

Study Intervention

1. What agent is used?
   1. Maraviroc
   2. Selzentry
   3. Celsentri
   4. Leronlimab
   5. Aplaviroc
   6. Vicriviroc
   7. Ancriviroc
   8. Other (please specify)
2. What is the dose (numeric value)?
3. What is the unit used for the dose?
4. What is the route of administration?
5. What was the agent prepared/diluted in?
6. When was the treatment administered?
   1. Pre-stroke
   2. Post-stroke
7. What is the timing in hours of intervention post-stroke (first adminsteration if applicable)?
8. Was the treatment administered at multiple timepoints?
   1. Yes (please specify the other timepoints in hours)
   2. No
9. How is time zero defined in the study?
10. Was the intervention paired with any other therapy (e.g. post-stroke rehabilitation paradigms)?
    1. Yes (please describe)
    2. No

Outcomes

1. Are motor behaviour outcomes (e.g. motor skills) being assessed? Please check all that apply
   1. Yes
      1. Neurological deficit score
      2. Rotarod
      3. Adhesive removal test
      4. Cylinder task
      5. Foot fault & paw placement tests (e.g. tapered beam, ladder, grid walking)
      6. Montoya staircase
      7. Reaching task (e.g. tray, pellet, pasta matrix)
      8. Corner test
      9. Staircase test
      10. Forelimb placing test
      11. Wire hanging test
      12. Other
   2. No
2. Are cognitive behavioural outcomes (e.g. cognitive skills) being assessed? Please check all that apply
   1. Yes
      1. Morris water maze
      2. Y-maze test
      3. Novel object recognition test
      4. Elevated plus maze
      5. Sucrose preference test
      6. Tail suspension test
      7. Open field test
      8. Forced swim test
      9. Other
   2. No
3. Was infarct volume/size measured?
   1. Yes
      1. What is the mean infarct size in mm3 or % of the hemisphere for the treatment animals?
      2. What is the mean infarct size in mm3 or % of the hemisphere for the control animals?
      3. What is the method for measuring infarct size? Please check all that apply
         1. Triphenyltetrazolium chloride (TTC)
         2. Cresyl violet (CV)
         3. Hematoxylin and eosin (H&E)
         4. Magnetic resonance imaging (MRI)
         5. Other (please specify)
      4. What is the (first, if applicable) time of the post-stroke measure of infarct size in days?
      5. Was infarct size measured multiple times?
         1. Yes (please list the other times in days)
         2. No
   2. No
4. How many animals are in the treatment and control groups?
5. What number of animals died for control and treatment groups?
6. What was the adverse effect (e.g. muscle fatigue, reduced mobility) for control and treatment groups
7. Were tissue outcomes (e.g. immunohistochemical staining, axonal neurofilament staining, pharmacogenetic techniques, axonal tracing techniques, genetic markers identification) measured or visualized?
   1. Yes, please explain
   2. No
8. Were brain imaging or biomarkers used to measure animal neural connectivity?
   1. Yes
      1. Diffusion tensor imaging (DTI)
      2. Resting state functional MRI (rsFMRI)
      3. Task-based functional MRI
      4. Other (please specify)
   2. No

Risk of Bias

“Yes” indicates low risk of bias; “no” indicates high risk of bias; and “unclear” indicates an unclear risk of bias. If one of the relevant signaling questions is answered with “no,” this indicates high risk of bias for that specific entry.

1. Was the allocation sequence adequately generated and applied?

*Did the investigators describe a random component in the sequence generation process such as:

- - - Referring to a random number table;
    - Using a computer random number generator.
    1. Yes
    2. No
    3. Unclear

Additional info:

Examples of a non-random approach:

- Allocation by judgement or by investigator’s preference;
- Allocation based on the results of a laboratory test or a series of tests;
- Allocation by availability of the intervention;
- Sequence generated by odd or even date of birth;
- Sequence generated by some rule based on animal number or cage number.

1. Were the groups similar at baseline or were they adjusted for confounders in the analysis?

*Was the distribution of relevant baseline characteristics balanced for the intervention and control groups?

- - 1. Yes
    2. No
    3. Unclear

*If relevant, did the investigators adequately adjust for unequal distribution of some relevant baseline characteristics in the analysis?

- - 1. Yes
    2. No
    3. Unclear

*Was the timing of disease induction adequate?

- - 1. Yes
    2. No
    3. Unclear

Additional info:

The number and type of baseline characteristics are dependent on the review question. Before starting their risk of bias assessment, therefore, reviewers need to discuss which baseline characteristics need to be comparable between the groups. In an SR investigating the effects of hypothermia on infarct size, for example, gender distribution, left ventricular weight and heart rate and blood pressure should be similar between the groups at the start of the study.

A description of baseline characteristics and/or confounders usually contains:

- The sex, age and weight of the animals
- Baseline values of the outcomes which are of interest in the study

Timing of disease induction:

In some prevention studies, the disease is induced after allocation of the intervention. For example, in an experiment on preventive probiotic supplementation in acute pancreatitis, pancreatitis is induced after allocation of the animals to the probiotic or control group. To reduce baseline imbalance, the timing of disease induction should be equal for both treatment groups.

Examples of adequate timing of disease induction:

- The disease was induced before randomization of the intervention.
- The disease was induced after randomization of the intervention, but the timing of disease induction was at random, and the individual inducing the disease was adequately blinded from knowing which intervention each animal received.

1. Was the allocation to the different groups adequately concealed during?

*Could the investigator allocating the animals to intervention or control group not foresee assignment due to one of the following or equivalent methods?

- Third-party coding of experimental and control group allocation Central randomization by a third party; Sequentially numbered opaque, sealed envelopes
  - 1. Yes
    2. No
    3. Unclear

Additional info:

Examples of investigators allocating the animals being possibly able to foresee assignments:

- Open randomization schedule
- Envelopes without appropriate safeguard
- Alternation or rotation
- Allocation based on date of birth
- Allocation based on animal number
- Any other explicitly unconcealed procedure of a non-random approach

1. Were the animals randomly housed during the experiment?

*Did the authors randomly place the cages or animals within the animal room/facility?

- - 1. Yes
    2. No
    3. Unclear
- Animals were selected at random during outcome assessment (use signaling questions of entry 6).

*Is it unlikely that the outcome or the outcome measurement was influenced by not randomly housing the animals?

- - 1. Yes
    2. No
    3. Unclear

The animals from the various experimental groups live together in one cage/pasture (e.g., housing conditions are identical).

Additional info:

Examples of investigators using a non-random approach when placing the cages:

- Experimental groups were studied on various locations (e.g., group A in lab A or on shelf A; Group B in Lab B or on shelf B).

1. Were the caregivers and/or investigators blinded from knowledge which intervention each animal received during the experiment?

*Was blinding of caregivers and investigators ensured, and was it unlikely that their blinding could have been broken?

- - 1. Yes
    2. No
    3. Unclear
- ID cards of individual animals, or cage/animal labels are coded and identical in appearance.
- Sequentially numbered drug containers are identical in appearance.
- The circumstances during the intervention are specified and similar in both groups (#).
- Housing conditions of the animals during the experiment are randomized within the room (use criteria of entry 4).

Additional info:

Examples of inappropriate blinding:

- Colored cage labels (red for group A, yellow group B)
- Expected differences in visible effects between control and experimental groups
- Housing conditions of the animals are not randomized within the room during the experiment; use criteria of entry 4
- The individual who prepares the experiment is the same as the one who conducts and analyses the experiment
- Circumstances during the intervention are not similar in both groups (#)
- Examples where circumstances during the intervention were not similar:
- Timing of administration of the placebo and exp drug was different.
- Instruments used to conduct experiment differ between experimental and control group (e.g., experiment about effects abdominal pressure; exp group receives operation and needle to increase pressure, while control group only has the operation).

**The relevance of the above-mentioned items depends on the experiment. Authors of the review need to judge for themselves which of the above-mentioned items could cause bias in the results when not similar. These should be assessed.

1. Were animals selected at random for outcome assessment?

*Did the investigators randomly pick an animal during outcome assessment, or did they use a random component in the sequence generation for outcome assessment?

- - 1. Yes
    2. No
    3. Unclear
- Referring to a random number table;
- Using a computer random number generator;
- Etc.

1. Was the outcome assessor blinded?

*Was blinding of the outcome assessor ensured, and was it unlikely that blinding could have been broken?

- - 1. Yes
    2. No
    3. Unclear
- Outcome assessment methods were the same in both groups.
- Animals were selected at random during outcome assessment (use signaling questions of entry 6).

*Was the outcome assessor not blinded, but do review authors judge that the outcome is not likely to be influenced by lack of blinding?

- - 1. Yes
    2. No
    3. Unclear

(e.g., mortality)

Additional info:

This item needs to be assessed for each main outcome.

1. Were incomplete outcome data adequately addressed? (*)

*Were all animals included in the analysis?

- - 1. Yes
    2. No
    3. Unclear

*Were the reasons for missing outcome data unlikely to be related to true outcome? (e.g., technical failure)

- - 1. Yes
    2. No
    3. Unclear

*Are missing outcome data balanced in numbers across intervention groups, with similar reasons for missing data across groups?

- - 1. Yes
    2. No
    3. Unclear

*Are missing outcome data imputed using appropriate methods?

- - 1. Yes
    2. No
    3. Unclear

1. Are reports of the study free of selective outcome reporting? (*)

*Was the study protocol available and were all of the study’s pre-specified primary and secondary outcomes reported in the current manuscript?

- - 1. Yes
    2. No
    3. Unclear

*Was the study protocol not available, but was it clear that the published report included all expected outcomes (i.e. comparing methods and results section)?

- - 1. Yes
    2. No
    3. Unclear

Additional info:

Selective outcome reporting:

  - Not all of the study’s pre-specified primary outcomes have been reported;

  - One or more primary outcomes have been reported using measurements, analysis methods or data subsets (e.g., subscales) that were not pre-specified in the protocol;

  - One or more reported primary outcomes were not pre-specified (unless clear justification for their reporting has been provided, such as an unexpected adverse effect);

  - The study report fails to include results for a key outcome that would be expected to have been reported for such a study.

1. Was the study apparently free of other problems that could result in high risk of bias? (*)

*Was the study free of contamination (pooling drugs)?

- - 1. Yes
    2. No
    3. Unclear

*Was the study free of inappropriate influence of funders?

- - 1. Yes
    2. No
    3. Unclear

*Was the study free of unit of analysis errors?

- - 1. Yes
    2. No
    3. Unclear

*Were design-specific risks of bias absent?

- - 1. Yes
    2. No
    3. Unclear

*Were new animals added to the control and experimental groups to replace drop-outs from the original population?

- - 1. Yes
    2. No
    3. Unclear

Additional info:

The relevance of the signaling questions (Table 3) depends on the experiment. Review authors need to judge for themselves which of the items could cause bias in their results and should be assessed.

Contamination/pooling drugs:

Experiments in which animals receive ‒ besides the intervention drug ‒ additional treatment or drugs which might influence or bias the result.

Unit of analysis errors:

- Interventions to parts of the body within one participant (i. e., one eye exp; one eye control).
- All animals receiving the same intervention are caged together, but analysis was conducted as if every single animal was one experimental unit.
- Design-specific risks of bias:
- Crossover design that was not suitable (intervention with no temporary effect, or the disease is not stable over time)
- Crossover design with risk of carry-over effect
- Crossover design with only first period data being available
- Crossover design with many animals not receiving 2nd or following treatment due to large number of drop-outs probably due to longer duration of study
- Crossover design in which all animals received same order of interventions
- Multi-arm study in which the same comparisons of groups are not reported for all outcomes (selective outcome reporting)
- Multi-arm study in which results of different arms are combined (all data should be presented per group)
- Cluster randomized trial not taking clustering into account during statistical analysis (unit of analysis error)
- Crossover design in which paired analysis of the results is not taken into account

Validity

1. Does the treatment effect vary with dose?
   1. Yes
   2. No
   3. Not applicable (only tested one dose)
   4. Unsure
2. Does the treatment remain effective when administered at clinically relevant delayed times?
   1. Yes (tested after 6 hours and the CCR5 antagonist was still effective)
   2. No (tested after 6 hours, but the CCR5 antagonist was not effective)
   3. Not applicable (not tested after 6 hours)
   4. Unsure
3. Does the treatment cause expected physiological effects?
   1. Yes
   2. No
   3. Unsure
4. Does the treatment penetrate the blood brain barrier?
   1. Yes
   2. No
   3. Unsure
5. What the tests done across multiple laboratories?
   1. Yes
   2. No
   3. Unsure
6. Was testing done on gyrencephalic species (non-human primates i.e. monkeys, dogs, pigs, etc.)
   1. Yes
   2. No
   3. Unsure
7. Did the preclinical testing of therapy occur during the awake phase for the animal model (during the dark phase for rodents i.e. was the test done during the nighttime)?
   1. Yes
   2. No
   3. Unsure

**********************************************************************************
